# Wnt5a Signaling in Normal and Cancer Stem Cells

**DOI:** 10.1155/2017/5295286

**Published:** 2017-04-12

**Authors:** Yan Zhou, Thomas J. Kipps, Suping Zhang

**Affiliations:** ^1^Medical Cancer Research Center, School of Medicine, Shenzhen University, Shenzhen 518060, China; ^2^Moores UCSD Cancer Center, University of California, San Diego, 3855 Health Sciences Dr., La Jolla, CA 92093, USA

## Abstract

Wnt5a is involved in activating several noncanonical Wnt signaling pathways, which can inhibit or activate canonical Wnt/*β*-catenin signaling pathway in a receptor context-dependent manner. Wnt5a signaling is critical for regulating normal developmental processes, including stem cell self-renewal, proliferation, differentiation, migration, adhesion, and polarity. Moreover, the aberrant activation or inhibition of Wnt5a signaling is emerging as an important event in cancer progression, exerting both oncogenic and tumor suppressive effects. Recent studies show the involvement of Wnt5a signaling in regulating normal and cancer stem cell self-renewal, cancer cell proliferation, migration, and invasion. In this article, we review recent findings regarding the molecular mechanisms and roles of Wnt5a signaling in stem cells in embryogenesis and in the normal or neoplastic breast or ovary, highlighting that Wnt5a may have different effects on target cells depending on the surface receptors expressed by the target cell.

## 1. Introduction

Stem cells, including embryonic stem cells and stem cells identified in adult tissues, have an ability to self-renew and to generate more differentiated progeny. The embryonic stem cell is derived from the inner cell mass of the blastocyst, is pluripotent, and can thus generate all the tissues of the body [1]. Stem cells in adult tissues reside in specialized niches, where they integrate various environmental and intrinsic signaling inputs that determine cell fate and maintain tissue homeostasis.

Wnt factors are a group of these signaling molecules that act on stem cells within the stem cell niche to help maintain their capacity for self-renewal. Wnt factors are known to bind to different receptors to transduce the canonical signaling pathway or noncanonical signaling pathway(s) that regulate diverse biological activities [2, 3]. The canonical Wnt signaling pathway is initiated when Wnt factors bind to Frizzled family receptors (Fzd) and low-density lipoprotein receptor-related protein 5/6 coreceptors (LRP5/6) to form complexes. This results in the recruitment of Axin and dishevelled (Dvl) to the plasma membrane and disruption of the *β*-catenin degradation complex, leading to accumulation of *β*-catenin in the cytoplasm, which then translocates to the nucleus, where it binds to T-cell factor (TCF)/lymphoid enhancer factor (LEF) family and activates the transcription of canonical Wnt target genes [4, 5]. In contrast, several Wnt factors activate *β*-catenin-independent pathways (noncanonical Wnt signaling pathways), known as the Wnt-Ca2^+^ pathway or planar cell polarity (PCP) pathway, which act to direct cell migration during embryogenesis [6–8].

Nineteen secreted cysteine-rich Wnt family glycoproteins have been identified in mice and humans. During earlier mammalian embryogenesis or maintenance of adult tissue homeostasis, these Wnt factors and their receptors are dynamically expressed to activate appropriate signaling, to ensure the right balance between proliferation and differentiation. Perturbation of Wnt signaling with aberrant expression of Wnt factors, their receptors, or downstream signaling molecules may lead to the development of several human cancers [6, 9, 10]. Because the tumor initiation capacity seems to be restricted to a small population of tumor cells that are endowed with the capacity for self-renewal and differentiation, they often are referred to as cancer stem cells (CSCs, or tumor initiation cells) [11–14]. More importantly, the biology of embryonic stem cells, normal stem cells, and CSCs is highly interrelated. This is evident from the fact that molecular signals (e.g., Wnt signaling) that define and maintain embryonic stem cell or adult normal stem cells are often aberrantly activated in tumor cells.

A major focus of this review is the role of Wnt5a signaling via different surface receptors in the regulation of stem cells in embryogenesis and in the normal or neoplastic breast or ovary, as there already are several excellent reviews that cover aspects of Wnt signaling in colon cancer [14, 15]. We summarized the latest advances on the signaling pathways that are activated by Wnt5a and that pose a conundrum for the rational design of drugs aimed at depleting the CSCs (or tumor initiation cells) within tumors while sparing the function of normal tissues.

## 2. Wnt5a Signaling Pathway

Wnt5a is a noncanonical Wnt ligand that is evolutionarily conserved and plays an important role in development. Homozygous Wnt5a knockout mice have perinatal lethality due to developmental defects [16]. Previous studies showed that Wnt5a can interact with Fzd2 and receptor tyrosine kinase-like orphan receptor 2 (ROR2) to activate Rac1 in a *β*-catenin-independent pathway. More recently, we demonstrated that Wnt5a induced heterooligermization of receptor tyrosine kinase-like orphan receptor 1/2 (ROR1/ROR2), which recruited and activated guanine exchange factors (GEFs), which in turn activated RhoA and Rac1, respectively, enhancing leukemia-cell chemotaxis and proliferation [17]. On the other hand, as another action of Wnt5a, it competed with Wnt3a for binding to Fzd2, thereby inhibiting Wnt3a-dependent LRP6 phosphorylation and *β*-catenin accumulation in vitro and in intact cells, inhibiting the capacity of Wnt3a to induce accumulation of *β*-catenin, and thereby inhibiting *β*-catenin-dependent Wnt signaling [18].

However, Wnt5a also could activate *β*-catenin-dependent pathway and induce secondary axis formation in Xenopus embryos that coexpressed the Fzd5 receptor [19, 20]. Subsequently, another study found that Wnt5a could inhibit canonical Wnt/*β*-catenin signaling in cells that expressed ROR2, but also could induce canonical Wnt/*β*-catenin signaling in cells that expressed Fzd4 and LRP5 [21]. Thus, a single Wnt5a ligand can have disparate effects on cells depending on receptor availability. Therefore, the cellular context dictates the effect of Wnt5a. This might account to the observation that the Wnt5a could exert either oncogenic or tumor suppressive effects in different cancers [18, 21–23].

## 3. Wnt5a Signaling in Embryonic Stem Cells

The Wnt pathway (triggered either by Wnt3a, Wnt5a, or Wnt6) also could be involved in the short-term maintenance of pluripotency of both mouse and human ES cells [24]. The first evidence for this was provided by a study of a pharmacological inhibitor of glycogen synthase kinase-3 (GSK3) that could modulate GSK3 activity, leading to activation of *β*-catenin canonical signaling and increased stability of c-Myc [25, 26]. Wnt5a and Wnt6 subsequently were found to be produced by embryonic fibroblast feeder cells, which could inhibit mouse ES cell differentiation in a serum-dependent manner. Direct activation of *β*-catenin, using constitutively active *β*-catenin (S33Y) without disturbing the upstream components of the Wnt/*β*-catenin, fully recapitulated the effect of Wnts on ES cells. Importantly, Wnt5a also is a potent inhibitor of *β*-catenin phosphorylation, thereby stabilizing *β*-catenin [5]. These data suggest that Wnt5a signals to stabilize *β*-catenin to further activate Wnt/*β*-catenin canonical signaling in mouse ES cells. Finally, the Wnt/*β*-catenin pathway can upregulate the mRNA for STAT3, a known regulator of ES cell self-renewal [27], suggesting a molecular mechanism whereby the Wnt/*β*-catenin pathway acts to prevent ES cell differentiation through convergence on the LIF/JAK-STAT pathway at the level of STAT3 [28]. However, what surface receptors are involved in the activation of Wnt5a/*β*-catenin signaling in ES cells and whether noncanonical Wnt signaling also could be activated by Wnt5a in ES cells remains unclear.

## 4. Wnt5a Signaling on Stem Cells in Normal Mammary Gland and Ovarian Tissues

A line of evidence supports the importance of Wnt in maintaining of mammary stem cells in mammary gland [29–31]. However, Wnt5a does not induce *β*-catenin/TCF transactivation activity in mammary gland tissue. The cells of the mammary gland are hyperproliferative in Wnt5a knockout mice, whereas in the presence of ectopic Wnt5a, ductal extension is inhibited [32]. These phenotypes are the inverse of the activation of canonical Wnt/*β*-catenin signaling. Therefore, Wnt5a may limit and control mammary gland growth by suppressing Wnt/*β*-catenin signaling. It is puzzling then that this inhibition of mammary gland growth was not observed in transgenic MMTV-Wnt5a strains [33].

In the *Drosophila* germarium, altered Wingless (Wg; the fly Wnt homologue) signaling occurs in ovarian germline stem cells (GSCs) through removal of its regulators such as dishevelled, armadillo, Axin, and shaggy; this causes GSC loss, influences follicle cell proliferation, and induces differentiation [34, 35]. Using conditional gene targeting for Wnt5a in ovarian granulosa cells (GC) results in the female subfertility associated with increased follicular atresia and decreased rates of ovulation. Further study found that Wnt5a regulates its target genes not by signaling via the Wnt/Ca^2+^ or planar cell polarity pathway, but rather by inhibiting the canonical pathway, causing both *β*-catenin and cAMP responsive element binding (CREB) protein levels to decrease via a glycogen synthase kinase-3*β*-dependent pathway in ovarian granulosa cells (GC). These data indicate that Wnt5a is required for normal ovarian follicle development and can antagonize gonadotropin responsiveness in granulosa cells by suppressing canonical Wnt/*β*-catenin signaling [36].

Collectively, Wnt5a is most likely to suppress *β*-catenin signaling in normal stem cells of mammary gland and ovarian tissues, although Wnt5a is able to activate *β*-catenin signaling in embryonic stem cells. Whether this difference is due to receptors that are differentially expressed in embryonic stem cells versus stem cells of normal mammary gland or the ovary needs to be further investigated.

## 5. Wnt5a Signaling in Breast Cancer Stem Cells

Wnt factors may affect various mammary epithelial cells and induce an expansion of stem cells during tumor progression [37–42]. Moreover, aberrant *β*-catenin expression was associated with basal, triple-negative breast cancers and poor clinical outcome [43, 44]. The occurrence of aberrant Wnt signaling in specific breast cancer subtypes is less likely dictated by activation caused by somatic mutations. Mutations have not been identified in *CTNNB1,* which encodes *β*-catenin, and mutations within *APC* have been identified in less than 20% of breast cancer patient samples [45]. Further investigation is needed to determine which Wnt ligands and receptors cause this aberrant Wnt/*β*-catenin signaling.

Recently, a study on MMTV-*Wnt1* mouse primary cells found that both recombinant Wnt3a and recombinant Wnt5a could enhance formation of mammospheres in vitro. Wnt5a-induced formation of mammospheres was not caused by an increase in canonical Wnt/*β*-catenin signaling, but was instead mediated by noncanonical Wnt signaling requiring the receptor tyrosine kinase ROR2 and the activity of the Jun N-terminal kinase, JNK, in mouse breast cancer cells [46]. This is consistent with the observation that patients with breast cancers that express ROR2 had a significantly shorter overall survival than those with tumors lacking expression of ROR2 [47]. However, silencing ROR2 in human breast cancer cell lines decreased both *β*-catenin-dependent and *β*-catenin-independent targets [47], suggesting that ROR2 may be involved in both *β*-catenin-dependent and *β*-catenin-independent Wnt signaling pathways. ROR1 predominately seems to be expressed by less well-differentiated tumors that have high potential for relapse and metastases and that also express markers associated with epithelial–mesenchymal transition (EMT) [48, 49]. Conversely, silencing ROR1 in human breast cancer cell lines could attenuate expression of genes associated with EMT and impair their migration/invasion capacity in vitro and their metastatic potential in vivo [49]. Very recently, Chien et al. reported that ROR1 expression might be an independent adverse prognostic factor in patients with triple-negative breast cancers [50]. Whether ROR1 can have a similar effect with ROR2 on breast cancer stem cell and what signaling pathway(s) are activated by ROR1 in breast cancer stem cells remains unknown.

A study on a mouse model of ErbB2-induced breast cancer found conflicting evidence on the effect of Wnt5a on human breast cancer stem cells. During ErbB2-induced mammary tumorigenesis, basal tumor-initiating cells (TIC), which exhibited enhanced tumorigenic capacity compared with the corresponding luminal progenitors, preferentially lost *Wnt5a* expression, as determined by transcript profiling analysis. Moreover, *Wnt5a* heterozygosity promoted tumor multiplicity and pulmonary metastasis. As a TGF*β* substrate, luminal cell-produced Wnt5a induced a feed-forward loop that activated SMAD2 in a RYK- and TGF*β*R1-dependent manner to limit the expansion of basal TIC in a paracrine fashion, a potential explanation for the suppressive effect of Wnt5a on mammary tumorigenesis [51]. In this mouse model, it remains uncertain whether canonical Wnt/*β*-catenin signaling was activated or if Wnt5a also inhibited canonical *β*-catenin signaling to suppress basal TIC expansion.

The Weinberg laboratory studied immortalized human mammary epithelial cells (mMECs) and found that both the canonical and noncanonical Wnt pathways cooperated with TGF*β* signaling in not only the maintenance, but intriguingly also the induction, of stem cell properties [52]. Moreover, only noncanonical Wnt ligands Wnt5a and Wnt16 were found upregulated in stem-like cells relative to non-stem-like cells [52]. Therefore, it is possible that Wnt5a or Wnt16 may activate either the canonical or noncanonical Wnt signaling pathway in a receptor context-dependent manner in human breast cancer stem cells.

## 6. Wnt5a Signaling in Ovarian Cancer Stem Cells

Similar to breast cancer, Wnt5a effect on ovarian cancer is also controversial. An early study of primary ovarian tumors (*n* = 130) that showed low levels of Wnt5a in ovarian cancer relative to normal ovary is predictive of a poor outcome [53]. Ectopic expression of Wnt5a inhibits the proliferation of human ovarian cancer cell line OVCAR5 both in vitro and in vivo orthotopic ovarian cancer mouse models. Mechanistically, ectopic expression of Wnt5a in OVCAR5 antagonizes canonical Wnt/*β*-catenin signaling and induces cellular senescence by activating the histone repressor A (HIRA)/promyelocytic leukemia (PML) senescence pathway [53].

In contrast, studies involving a large number of patients found that upregulation of Wnt5a was associated with a relatively poor prognosis [54–58]. Compared with the frequency of Wnt1 expression in ovarian cancer, Wnt5a was more frequently found in malignant epithelial ovarian cancer patients (80% out of a total 38) [54]. Of note, patients with ovarian cancers that express high levels of both Wnt1 and Wnt5a had a significantly lower probability of long-term survival than patients with ovarian cancers that did not express Wnt1 or Wnt5a. Furthermore, Wnt5a is prevalent in ascites fluid obtained from women with ovarian cancer [55], suggesting that it contributes to the ovarian tumor microenvironment. This is consistent with the observation that high levels of Wnt5a are associated with increased risk for metastasis [55].

Activating mutations in the canonical Wnt pathway is rare in ovarian cancer, with the exception of endometrioid ovarian cancers [59]. The contribution of canonical Wnt signaling to progression of ovarian cancer remains unknown. Conversely, studies on noncanonical Wnt/*β*-catenin signaling show that Wnt5a may regulate ovarian cancer EMT, migration, or metastasis via noncanonical signaling pathways [57, 60]. Consistent with this notion, recent studies have found that expression of ROR1, a receptor of Wnt5a, was highly expressed by high-grade and less-differentiated ovarian cancers and associated with a relatively short disease-free survival and overall survival compared to ovarian cancers that did not express ROR1 [61–63]. Ovarian cancers that express high levels of ROR1 had gene expression signatures associated with ovarian CSCs [62]. Furthermore, tumor-cell expression of ROR1 apparently correlates with the expression of ALDH1 and the capacity to form tumor spheroids in vitro (both markers of CSC). Treating primary ovarian patient-derived xenograft (PDX) tumor cells, which express high levels of ROR1 with an anti-ROR1 mAb (UC-961 or cirmtuzumab), inhibited spheroid formation and migration in vitro and engraftment and re-engraftment in immune-deficient mice, indicating that ROR1 may influence ovarian cancer stem cell self-renewal. Further studies are needed to determine if Wnt5a is responsible for the influence that ROR1 apparently has on the biology of ovarian cancer stem cells.

## 7. Concluding Remarks

Wnt5a may play an important role in embryonic stem cell and organ development. However, the role of Wnt5a in cancer stem cells is varied and complex. It may suppress or promote tumor progression. To elucidate molecular mechanisms that drive altered cellular behavior, additional research on tissue-specific expression of specific receptors and coreceptors is needed. In particular, a more detailed understanding of the complex cross-talk between Wnt5a and specific receptors that are expressed in embryonic stem cells and that may be re-expressed or reactivated in cancer stem cells, but not in normal somatic tissues (e.g., ROR1 and ROR2), may enable development of specific inhibitors that block aberrant signaling and thereby favorably impact patient survival.
