# Corrigendum to “Wnt5a Signaling in Normal and Cancer Stem Cells”

**DOI:** 10.1155/2017/3467360

**Published:** 2017-09-27

**Authors:** Yan Zhou, Thomas J. Kipps, Suping Zhang

**Affiliations:** ^1^Medical Cancer Research Center, School of Medicine, Shenzhen University, Shenzhen 518060, China; ^2^Moores UCSD Cancer Center, University of California, San Diego, 3855 Health Sciences Dr., La Jolla, CA 92093, USA

In the article titled “Wnt5a Signaling in Normal and Cancer Stem Cells” [1], the Acknowledgments section should be corrected as follows:

“This study was supported by the Breast Cancer Research Foundation (NIH Grant P01-CA081534), the California Institute for Regenerative Medicine, the National Natural Science Foundation of China (NSFC, Grant no. 81672912), and the Science and Technology Foundation of Shenzhen, China (Shenzhen Peacock Innovation Team Project, Grant no. KQTD20140630100658078).”
